# Photoaging Protective Effects of *Ranunculus bulumei* Methanol Extract

**DOI:** 10.1155/2020/1761785

**Published:** 2020-04-03

**Authors:** Yo Han Hong, Ji Hye Kim, Jae Youl Cho

**Affiliations:** Department of Integrative Biotechnology, Sungkyunkwan University, Suwon 16419, Republic of Korea

## Abstract

Ultraviolet B (UVB) radiation is the main cause of photoaging processes including cellular senescence, skin drying, collagen degradation, melanogenesis, and inflammation. These responses occur because UVB induces a change in expression of aging-related genes through regulation of signal pathways such as that of mitogen-activated protein kinases- (MAPKs-) activator protein 1 (AP-1). *Ranunculus bulumei*, which is used as an herb in Indonesia, belongs to the Ranunculaceae family, which has been reported to perform various physiological effects including antioxidant and anti-inflammation. However, data on the pharmaceutical and cosmeceutical utility of *Ranunculus bulumei* have not been reported. Therefore, we evaluated the antiaging efficacy of RB-ME, a methanol extract of *Ranunculus bulumei*. Rb-ME attenuated MMP9 and COX-2 gene expression but enhanced SIRT1 and type-1 collagen in UVB-irradiated HaCaT cells. Rb-ME regulated these gene expressions through inhibition of p38 phosphorylation and inactivation of AP-1. In addition, mRNA expression of HAS-2 and -3, which are involved in skin hydration, was elevated in Rb-ME-treated HaCaT cells. Rb-ME also inhibited melanogenesis by suppression of tyrosinase, MITF, and TYRP-1 mRNA in B16F10 cells under *α*-MSH treatment. Taken together, these results indicate that Rb-ME has a protective effect on some UVB-induced skin photoaging events such as inflammation, collagen degradation, cellular senescence, skin drying, and melanin production through inhibition of the p38-AP-1 signal cascade, indicating that Rb-ME can be used as an active ingredient for antiaging cosmetics.

## 1. Introduction

Skin is an anatomic barrier that protects our body from diverse pathogens and external stress. Ultraviolet (UV) radiation is a major cause of skin damage and has a wide range of wavelengths from 100 to 400 nm (UVA; 100∼280 nm, UVB; 280∼320 nm, UVC; 320∼400 nm). Especially, UVB is one of the inducers of various photoaging processes such as cellular senescence, skin drying, collagen degradation, melanogenesis, and inflammation by inducing alterations in expression of various aging-related molecules. For example, UV reduces expression and activity of epidermal Sirtuin (SIRT), an antiaging marker in cellular senescence [1]. The synthesis of type-1 procollagen (Col1a1), which is necessary for skin elasticity, is also decreased by UV irradiation [2]. On the other hand, the expression of MMP, which produces wrinkles through collagen degradation, or COX-2, which causes inflammation, is increased by UV treatment [3–5]. In addition, UV acts directly on keratinocytes to increase melanogenesis through upregulation of receptor expression for melanocyte-stimulating hormone (MSH) and induction of tyrosinase gene expression, which leads to pigmentation and age spots [6]. In aged skin, the expression of skin barrier factors FLG and TGM and the skin moisturizing factor hyaluronic acid synthase (HAS-1, 2, 3) is decreased, resulting in dry skin and loss of skin elasticity [7, 8].

Activator protein 1 (AP-1) was initially identified as a transcription factor that regulates basal gene expression [9] but has subsequently been reported to be activated by a variety of stimulants to regulate expression of target genes [10]. As AP-1 is activated by oncoproteins, such as v-Src and Ha-Ras, the importance of AP-1 in cancer development has been actively studied [11]. In addition, the response of AP-1 to various cytokines and pathogen infections suggests that AP-1 plays an essential role in immune responses, including inflammation [12]. UV is another stimulant of AP-1 and is known to induce and activate phosphorylation of AP-1 through activation of mitogen-activated protein kinases (MAPKs). There are three types of MAPKs (ERK, JNK, and p38), and JNK and p38 are phosphorylated by UV irradiation [13]. The activated MAPK signals ultimately induces homodimer or heterodimer formation of AP-1 subunits belonging to the Jun and Fos families through phosphorylation, leading to activity as transcription factors [13].

*Ranunculus bulumei* is a flowering plant belonging to the Ranunculaceae family, which is classified into 43 genera, the largest of which is *Ranunculus*. *Ranunculus* has a total of 600 species that have been reported to exhibit various medical effects. For example, *Ranunculus muricatus* L. has been used as a folk remedy for treating heart disease, cancer, and dental diseases [14–16]. *Ranunculus arvensis* L. has been utilized to cure arthritis, asthma, hay fever, rheumatism, psoriasis, and gut disease [17]. In addition, *Ranunculus muricatus* L. and *Ranunculus arvensis* L. methanol extracts were found to contain potent antioxidants [14, 17, 18]. In addition, it has been reported that methanol extracts of *Ranunculus peltatus subs* were effective against contact dermatitis [19], and *Ranunculus constantinopolitanus* has anti-inflammatory activities [20]. However, to our knowledge, there is no report on the activity of *Ranunculus bulumei*. Therefore, in this study, we aim to observe the activity of *Ranunculus bulumei* methanol extract (Rb-ME) in terms of skin antiaging properties. To do this, we examine the protective activity of Rb-ME on dry skin and UVB-induced photoaging processes including cellular senescence, collagen degradation, and melanogenesis.

## 2. Materials and Methods

### 2.1. Materials

Phorbol-12-myristate-13 acetate (PMA) and 3-(4-5-dimethylthiazol-2-yl)-2-5-diphenyltetrazolium bromide (MTT) were obtained from Sigma Chemical Co. (St. Louis, MO, USA). The luciferase construct harboring AP-1 and Col1A1 promoter-binding sites was used as reported earlier [21, 22]. TRIzol reagent was purchased from Molecular Research Center (Montgomery, OH, USA). Fetal bovine serum and Dulbecco's modified eagle's media (DMEM) were purchased from Gibco (Grand Island, NY, USA). The cell lines used in the present experiments (HaCaT, HEK293, and B16F10 cells) were obtained from ATCC (Rockville, MD, USA). All other chemicals were obtained from Sigma Chemical Co. (St. Louis, MO, USA). Plasmid constructs driving the expression of Smad3 were used as reported previously [23]. Antibodies against phosphorylation-specific and total forms of ERK, JNK, p38, and *β*-actin were purchased from Cell Signaling Technology (Beverly, MA, USA).

### 2.2. Cell Culture

Human keratinocyte HaCaT cells and mouse melanoma B16F10 cells were cultured in DMEM supplemented with 10% fetal bovine serum and 1% antibiotics (penicillin and streptomycin) in a CO_2_ incubator at 37°C. For experiments, cells were seeded in 6-well plates at a density of 1.0 × 10^6^ cells/well with fresh complete culture medium.

### 2.3. Preparation of *Ranunculus bulumei* Methanol Extract

*Ranunculus bulumei* methanol extract (Rb-ME) was prepared by a general protocol provided by the Korea Research Institute of Bioscience & Biotechnology International Biological Material Research Center. The aerial parts of *Ranunculus bulumei* (54 g) were soaked for extraction in 1 L of 99.9% (v/v) methanol with repeated sonication (15 min) and resting (2 h) for 3 days at 45°C. The resultant product was filtered with cottons and concentrated by using a rotary evaporator (N-1000SWD, EYELA) under reduced pressure at 45°C. Finally, 7.4 g of Rb-ME was obtained by freeze-drying. The final Rb-ME was a dark brown powder and was stored at −5°C until use.

### 2.4. Drug Treatment

A stock solution of Rb-ME was prepared in dimethyl sulfoxide (DMSO) at a concentration of 100 mg/mL. Target concentrations (0 to 200 or 0 to 400 *μ*g/mL) were achieved by dilution with culture medium, according to the activity of Rb-ME in different assay systems and cells.

### 2.5. Cell Viability Assay

HaCaT and B16F10 cells were seeded onto 96-well plates at a density of 1.0 × 10^5^ cells/well with fresh complete culture medium. To test the cytotoxicity of Rb-ME alone, cells were treated with Rb-ME (12.5 to 400 *μ*g/mL). To test the effect of Rb-ME on UVB-induced cytotoxicity, cells were irradiated with UVB (30 mJ/cm^2^) and then cultured in complete culture medium with Rb-ME (50 or 100 *μ*g/mL) for a further 24 h. Cell viability was determined with a conventional MTT assay [24].

### 2.6. UVB Irradiation

Cells were irradiated in 6-well plates using a UVB lamp (Bio-Link BLX-312, VILBER LOURMAT, France) with an emission wavelength peak of 312 nm. Before UVB irradiation, culture medium was replaced with 1 ml of phosphate-buffered saline (PBS) per well. After removing the plate lid, cells were irradiated at 30 mJ/cm^2^ [25]. After UVB irradiation, PBS was replaced with complete culture medium with the appropriate compound treatments prior to harvesting.

### 2.7. HPLC Analysis

The concentrations of Rb-ME were quantified by HPLC as described previously [26].

### 2.8. Plasmid Transfection and Luciferase Reporter Gene Assay

For the luciferase reporter gene assay, HEK293 cells (1.0 × 10^5^ cells/well in 24-well plates) were transfected with 0.8 *μ*g/mL of plasmids driving the expression of *β*-galactosidase, AP-1-luc, Col1A1-luc, and FLAG-Smad3. Cells were transfected using the polyethyleneimine (PEI) method [27] and then incubated for 24 h. Finally, HEK293 cells were treated with Rb-ME (50 or 100 *μ*g/mL) and PMA (100 nM) for a further 24 h.

### 2.9. Analysis of mRNA Levels by Reverse Transcriptase-Polymerase Chain Reaction (RT-PCR)

To quantify cytokine mRNA expression levels, HaCaT cells were treated with Rb-ME (50 or 100 *μ*g/mL), SB203580 (20 *μ*M), SP600125 (20 *μ*M), or U0126 (20 *μ*M) after UVB (30 mJ/cm^2^) irradiation. Total RNA was then isolated with TRIzol reagent according to the manufacturer's instructions. RT-PCR was performed as described previously [28]. Primers used in this study are listed in Table 1.

### 2.10. Immunoblotting

HaCaT cells were UV irradiated with or without Rb-ME (50 and 100 *μ*g/ml) for 24 h. To prepare whole lysates, cells were collected with trypsin, washed with cold 1x PBS, and lysed in lysis buffer (50 mM Tris-HCL, pH 7.5, 20 mM NaF, 25 mM *β*-glycerol phosphate, pH 7.5, 120 mM NaCl, 2% NP-40, 2 *μ*g/mL leupeptin, 2 *μ*g/mL aprotinin, 2 *μ*g/mL pepstatin A, 100 *μ*M Na_3_VO_4_, 1 mM benzamide, 100 *μ*M PMSF, and 1.6 mM pervanadate) by rotating for 30 min at 4°C. The lysates were used after clarification by centrifugation at 16,000*g* for 10 min at 4°C. Total lysates prepared from HaCaT cells were subjected to western blot analysis of the total and phospho-forms of JNK, ERK, p38, and *β*-actin. Immunoreactive bands were visualized as described previously [29].

### 2.11. Tyrosinase Assay

For the tyrosinase assay, 50 *μ*l of L-DOPA (6 mM) dissolved in potassium phosphate buffer (50 mM, pH 6.8), 50 *μ*l of dimethyl sulfoxide (DMSO) with or without Rb-ME (200, 400, or 800 *μ*g/mL), and kojic acid (200 *μ*M) dissolved in potassium phosphate buffer were mixed at room temperature for 15 min. Mushroom tyrosinase (100 units/mL) dissolved in potassium phosphate buffer was then added to the mixture. The absorbance of the mixture at 475 nm was immediately measured using a multidetection microplate reader.

### 2.12. Melanin Formation and Secretion Test

For the melanin formation assay, B16F10 cells (1.0 × 10^5^ cells/well in 12-well plates) were treated with *α*-MSH (100 nM), target concentrations of Rb-ME, or arbutin (1 mM) for 48 h [30]. Melanin secretion was then assessed by measuring the absorbance of the culture medium at 475 nm using a multidetection microplate reader. For melanin content analysis, cells were lysed with 20 *μ*L cell lysis buffer (Tris-HCl (50 mM, pH 7.5), NaF (20 mM), *β*-glycerolphosphate (25 mM, pH 7.5), NaCl (120 mM), and 2 % NP-40 in distilled water). The lysed pellets were dissolved in 90 *μ*L NaOH (1 M) containing 10 % DMSO for 30 min at 55°C, after which the absorbance of the resulting solutions was measured at 405 nm.

### 2.13. Statistical Analysis

All data are presented as mean ± standard deviation, and each experiment consisted of three or four replications. The Mann–Whitney *U* test was used to analyze the statistical difference between groups. A *p* value <0.05 was regarded as statistically significant. All statistical tests were performed using SPSS software (version 22.0, 2013; IBM Corp., Armonk, NY, USA).

## 3. Results

### 3.1. Measurement of Cytotoxicity and Flavonoid Profile of Rb-ME

**Figure 1:**
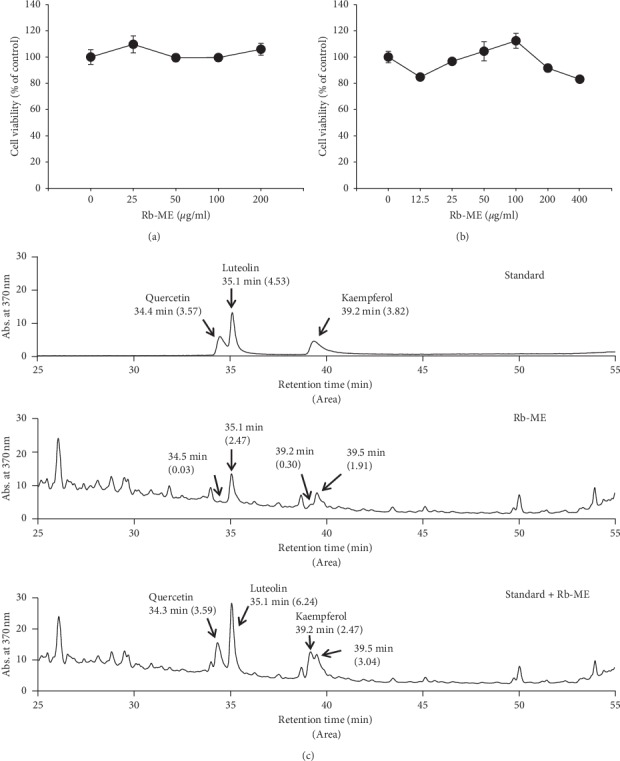
Effects of Rb-ME on the viability of HaCaT and B16F10 cells. HaCaT (a) and B16F10 cells (b) were treated with various concentrations (0 to 400 *μ*g/mL) of Rb-ME and then incubated for 24 or 48 h. Cell viability was measured using the MTT assay. (c) The phytochemical profiles of kaempferol, luteolin, and quercetin in Rb-ME were analyzed by HPLC.

To test the effects of Rb-ME on cell viability, we performed MTT assay using human keratinocyte cell line HaCaT and mouse melanoma cell line B16F10. We confirmed that Rb-ME was not cytotoxic up to 200 *μ*g/mL in HaCaT cells and 400 *μ*g/mL in B16F10 cells (Figures 1(a) and 1(b)). The levels of quercetin, luteolin, and kaempferol in Rb-ME were determined by high-performance liquid chromatography (HPLC). Quercetin, luteolin, and kaempferol were measured at 34.4 min, 35.1 min, and 39.2 min, respectively, in Rb-ME (Figure 1(c)).

### 3.2. Effect of Rb-ME on UVB Irradiation-Induced Cell Damage in HaCaT Cells

**Figure 2:**
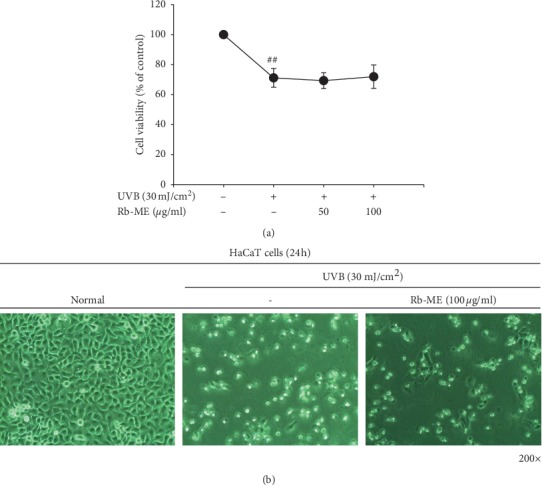
No effect of Rb-ME was noted on the UVB irradiation-induced damage in HaCaT cells. (a) HaCaT cells were irradiated with UVB (30 mJ/cm^2^) and then treated with or without Rb-ME (50 or 100 *μ*g/mL) for 24 h. Cell viability was measured using the MTT assay. (b) Images of HaCaT cells treated with or without Rb-ME (100 *μ*g/mL) for 24 h were obtained with a digital camera after irradiation with UVB (30 mJ/cm^2^). Magnification of the images is 200 times. ^##^*p* < 0.01 compared to the normal group.

To investigate the protective effect of Rb-ME on cell damage in UVB-irradiated HaCaT cells, we performed MTT assay. As shown in Figure 2(a), UVB irradiation (30 mJ/cm^2^) reduced the survival rate of HaCaT cells by 60%, and Rb-ME (50 or 100 *μ*g/ml) did not restore cell viability. Since cell death is accompanied by cell morphological change, we observed the effect of Rb-ME on alteration of cellular shape in UV-treated HaCaT cells. Rb-ME (50 or 100 *μ*g/ml) did not regulate UVB-induced cell morphological change (Figure 2(b)), indicating that Rb-ME is independent of regulation of cell damage under UV irradiation.

### 3.3. Effects of Rb-ME on Photoaging Responses in UVB-Irradiated HaCaT Cells

**Figure 3:**
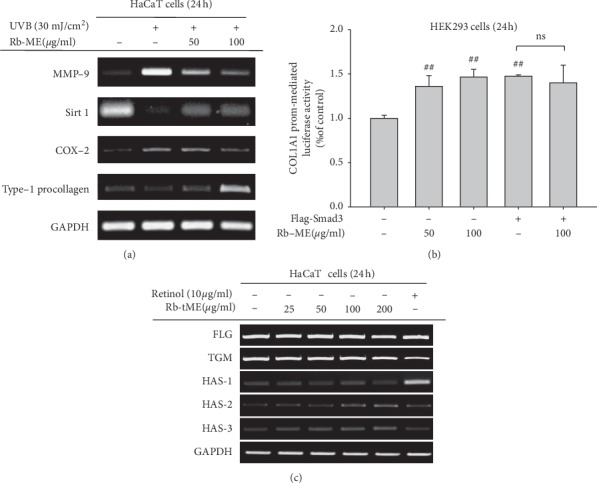
Effects of Rb-ME on photoaging responses in UVB-irradiated HaCaT cells. (a) HaCaT cells were irradiated with UVB (30 mJ/cm^2^) and then treated with or without Rb-ME (50 or 100 *μ*g/mL) for 24 h The mRNA levels of MMP-9, COX-2, SIRT1, and type-1 procollagen were analyzed by RT-PCR. (b) The promoter-binding activity of the transcription factor Col1A1 was determined using a luciferase reporter gene assay. HEK293 cells were transfected with plasmids driving the expression of Col1A1-Luc (1 *μ*g/mL), *β*-gal (as a transfection control), and FLAG-Smad3 (1 *μ*g/mL) and then treated with or without Rb-ME (50 or 100 *μ*g/mL) for 24 h. Luciferase activity was measured using a luminometer. (c) HaCaT cells were treated with Rb-ME (25 to 200 *μ*g/mL) or retinol (10 *μ*g/mL) for 24 h. The mRNA levels of FLG, TGM, HAS-1, HAS-2, and HAS-3 were analyzed by RT-PCR. ^##^*p* < 0.01 compared to the normal group or compared to the control group.

To determine the effects of Rb-ME on the photoaging process, HaCaT cells were treated with Rb-ME (50, 100 *μ*g/ml) after UVB (30 mJ/cm^2^) irradiation for 24 h. We confirmed mRNA levels of MMP-9, SIRT1, COX-2, and type-1 procollagen. UVB irradiation upregulated the mRNA levels of MMP-9 and COX-2 but suppressed the mRNA levels of SIRT1 and type-1 procollagen. Rb-ME (50 or 100 *μ*g/ml) dose-dependently decreased mRNA levels of MMP-9 and COX-2 but increased mRNA levels of SIRT1 and type-1 procollagen in UVB-irradiated HaCaT cells (Figure 3(a)). To elucidate whether Rb-ME directly regulates collagen synthesis at the transcriptional level, we conducted Col1a1 luciferase assay in the HEK293 cells. Rb-ME (50 or 100 *μ*g/ml) dose-dependently increased Col1a1 promotor activity up to 1.5 fold, although the increase level of luciferase activity by this extract was similar to that under Smad3 cotransfection conditions (Figure 3(b)). Also, there was no difference of luciferase activity between Smad3 alone and Rb-ME (100 *μ*g/ml) under Smad3 transfection conditions (Figure 3(b)).

Next, the moisturizing capacity of Rb-ME was evaluated by observing the expression alterations of skin barrier components, such as FLG and TGM, and HASs that synthesize HA. Of these molecules, Rb-ME (50 or 100 *μ*g/ml) only increased the HAS-2 and HAS-3 gene expression in UVB-irradiated HaCaT cells (Figure 3(c)). Our results suggest that Rb-ME has protective effects against UVB-induced photoaging processes.

### 3.4. Effect of Rb-ME on AP-1 Signaling in HaCaT Cells

**Figure 4:**
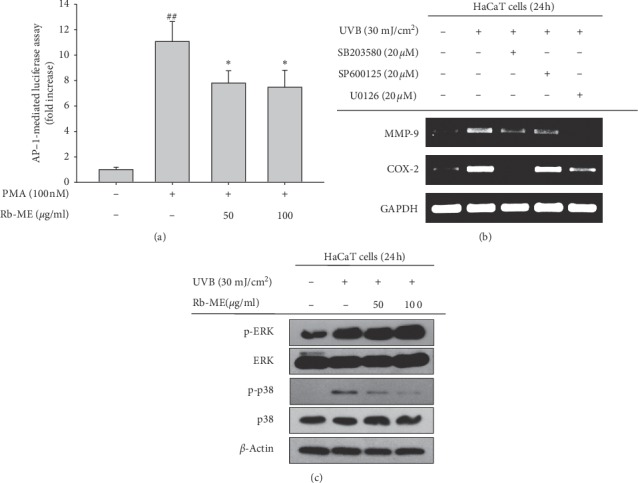
Effect of Rb-ME on the AP-1 signaling pathway in UV-irradiated HaCaT cells. (a) The promoter-binding activity of the transcription factor AP-1 was determined using a reporter gene assay. HEK293 cells were transfected with plasmids driving the expression of AP-1-Luc (1 *μ*g/mL) and *β*-gal (as a transfection control) in the presence or absence of PMA (100 nM) and Rb-ME (50 or 100 *μ*g/mL) for 24 h. (b) The effects of MAPK inhibitors (SB203580 (a p38 inhibitor, 20 *μ*M), SP600125 (a JNK inhibitor, 20 *μ*M), and U0126 (an ERK inhibitor, 20 *μ*M)) on the mRNA levels of MMP-9 and COX-2 in UVB-irradiated HaCaT cells were determined by RT-PCR. (c) The protein levels of phospho- and total forms of JNK, ERK, p38, and *β*-actin in whole cell lysates of UV-irradiated HaCaT cells treated with or without Rb-ME (50 or 100 *μ*g/mL) were determined by immunoblotting. ^##^*p* < 0.01 compared to the normal group and ^*∗*^*p* < 0.05 compared to the control group.

Previous studies reported that UVB irradiation activates the AP-1 signaling pathway [31]. In addition, AP-1 is involved in regulation of MMP-9 and COX-2 expression under UV-irradiation conditions [32, 33]. Therefore, we assessed whether Rb-ME can regulate AP-1 signaling. Rb-ME (50 or 100 *μ*g/ml) significantly inhibited PMA-induced AP-1 luciferase activity (Figure 4(a)). Next, we used MAPK inhibitors (SB203580; a p38 inhibitor, SP600125; a JNK inhibitor, and U0126; an ERK inhibitor) to investigate which molecules in MAPKs, the upstream signals of AP-1, are involved in regulation of photoaging markers MMP-9 and COX-2. All three inhibitors remarkably inhibited the mRNA level of MMP-9 on the UVB-irradiated HaCaT cells, and expression of COX-2 mRNA was diminished by SB2030580 and U0126 (Figure 4(b)), implying that p38 and ERK can positively regulate the expression of MMP-9 and COX-2 under UVB irradiation. Finally, we confirmed total and phospho-forms of MAPKs on the UVB-irradiated HaCaT cells without or with Rb-ME (50 or 100 *μ*g/ml). In our experimental conditions, only phosphorylation of ERK and p38 was increased by UV treatment, and Rb-ME dose-dependently suppressed UVB-induced phosphorylation of p38 (Figure 4(c)). These results imply that Rb-ME is involved in the expression of aging-related molecules through p38-specific regulation under UV-irradiated condition.

### 3.5. Antimelanogenesis Effect of Rb-ME in MSH-Treated B16F10 Cells

**Figure 5:**
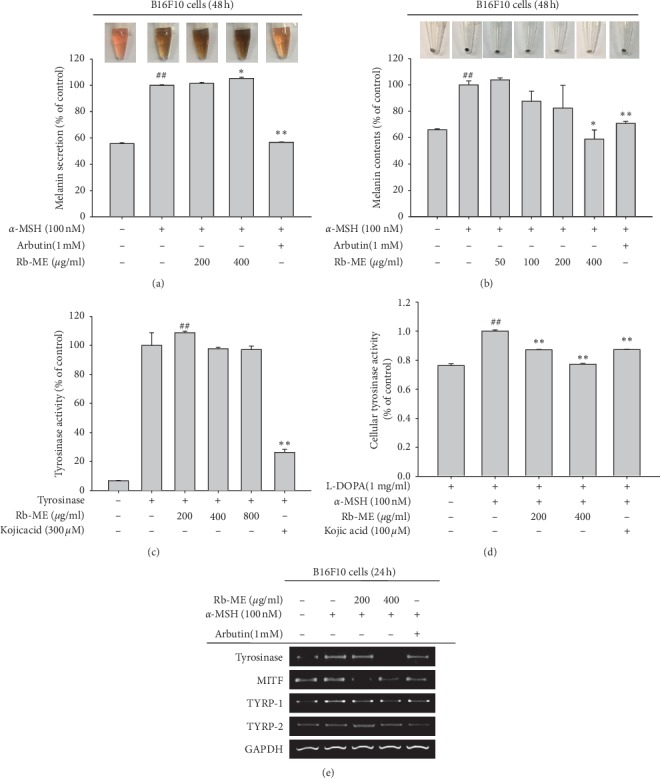
Antimelanogenesis effect of Rb-ME in *α*-MSH-treated B16F10 cells. (a, b) The levels of melanin secretion and contents in B16F10 cells treated with *α*-MSH (100 nM) in the presence or absence of Rb-ME (50 to 400 *μ*g/mL) or arbutin (1 mM) for 48 h were then determined. (c, d) The effect of Rb-ME (200 to 800 *μ*g/mL) or kojic acid (100 or 300 *μ*M) on mushroom tyrosinase activity was analyzed by measuring the activity of purified tyrosinase. (e) B16F10 cells treated with *α*-MSH (100 nM) in the presence or absence of Rb-ME (200 or 400 *μ*g/mL) or arbutin (1 mM) for 24 h. The mRNA levels of tyrosinase, MITF, TYRP-1, and TYRP-2 were then determined by RT-PCR. ^##^*p* < 0.01 compared to the normal group. ^*∗*^*p* < 0.05 compared to the control group and ^*∗∗*^*p* < 0.01 compared to the control group.

To determine the antimelanogenesis effect of Rb-ME, we examined whether Rb-ME reduced *α*-MSH-induced melanogenesis on B16F10 cells. Arbutin (1 mM) and kojic acid (100 or 300 *μ*M) were used as positive control drugs, since both are melanogenesis inhibitors through suppression of tyrosinase activity. The degree of melanin secretion and content was increased about 2-fold in *α*-MSH-treated B16F10 cells (Figures 5(a) and 5(b)). Rb-ME (50 or 100 *μ*g/*μ*l) had no significant effect on melanin secretion (Figure 5(a)), but melanin content was dose-dependently inhibited by Rb-ME treatment up to 400 *μ*g/ml (Figure 5(b)), indicating that Rb-ME has a function in melanin production but not secretion. It has been reported that tyrosinase is involved in melanin production [34]; therefore, we confirmed whether Rb-ME can modulate tyrosinase activity. Rb-ME did not directly inhibit mushroom tyrosinase activity (Figure 5(c)) but significantly inhibited *α*-MSH-induced cellular tyrosinase activity in B16F10 cells (Figure 5(d)). Then, we analyzed the effect of Rb-ME on the melanogenesis pathway. Thus, we examined mRNA levels of tyrosinase, MITF, TYRP-1, and TYRP-2. The mRNA levels of tyrosinase, MITF, and TYRP-1 were increased in *α*-MSH-induced B16F10 cells, while Rb-ME (200 or 400 *μ*g/ml) decreased mRNA levels of tyrosinase, MITF, and TYRP-1 in *α*-MSH-induced B16F10 cells (Figure 5(e)). These results suggest that Rb-ME suppresses melanogenesis through regulation of gene expression of tyrosinase, MITF, and TYRP-1.

## 4. Discussion

In this study, we evaluated the antiaging capacity of Rb-ME using HaCaT cells, a human keratinocyte cell line, and B16F10 cells, a mouse melanoma cell line. The effect of Rb-ME on cell death, a representative factor for skin aging, was examined under UV irradiation. Next, the effects of Rb-ME on the expression of aging-related proteins MMPs, COX-2, SIRT1, and type-1 collagen and on the signals associated with these molecules' mRNA expression were assessed under UV irradiation. We also studied whether Rb-ME can increase the expression of skin barrier components that weaken as aging progresses. Finally, the effect of Rb-ME on melanogenesis, a representative phenomenon of aging, was studied.

First, we performed component analysis of Rb-ME through flavonoid profiling. Flavonoids are phenolic natural substances and plant secondary metabolites found in a variety of vegetables and fruits [35], numbering more than 5000. Among them, quercetin, luteolin, and kaempferol are the most studied compounds [36, 37], so the presence of these three components was evaluated. As a result, it was observed that Rb-ME contains quercetin, luteolin, and kaempferol. Quercetin has activities such as oxygen radical scavenging, inhibition of lipid peroxidation, and metal ion chelation [38]. Kaempferol has antioxidant, anti-inflammatory, anticancer, and cardioprotective properties [39–41]. Lutein also has a strong antioxidant effect and is reported to be effective against a variety of diseases, including cancer and vascular diseases, and particularly beneficial to eye health [42, 43]. This evidence suggests the Rb-ME containing quercetin, kaempferol, and luteolin has protective activity against UVB-induced skin damages, such as cell death and photoaging, and melanogenesis.

As we expected, Rb-ME showed antiaging activity by decreasing the expression of photoaging-related molecules such as MMP-9 [3] and COX-2 [44], which were increased by UV irradiation. Rb-ME also reduced the Luc activity of AP-1, known as the major regulators of MMP-9 and COX-2, and these results led us to study the role of MAPK, an upstream regulator of AP-1, in the mechanism of Rb-ME activity. We observed that all three MAPKs are essential for MMP-9 expression, and p38 and ERK are needed for expression of COX-2 under UV treatment. Interestingly, Rb-ME reduced only the phosphorylation of p38 among MAPKs, which indicates that Rb-ME exhibits antiaging activity through regulation of p38-AP-1 signaling. Based on the report that ROS increased with UV acts as a secondary messenger to activate MAPK [45], it is speculated that the radical scavenging activity of the flavonoids present in Rb-ME might have reduced MAPK.

According to our results, UV seems to decrease skin elasticity not only by increasing MMP-9 but also directly by decreasing type-1 procollagen expression. Intriguingly, Rb-ME restored the expression of type-1 procollagen reduced by UV and increased the Luc activity of COL1A1. However, Rb-ME did not affect the Luc activity of Smad-induced Col1A1, implying that Rb-ME might control the Smad pathway. It has been reported that elevated AP-1 reduces the synthesis of type-1 procollagen via impairing transforming growth factor-*β* (TGF-*β*)/Smad signaling [46, 47]. Therefore, we concluded that Rb-ME might not only stimulate the synthesis of Col1A1 through the Smad pathway but also prevent degradation of procollagen due to inhibition of the p38/AP-1/MMP-9 axis. In addition, the expression of SIRT1 [48], which serves to protect cells from ROS, was reduced by Rb-ME. Overall, Rb-ME appears to exhibit antiaging efficacy by restoring the expression of aging-related genes altered by UV.

In aged skin, increased expression of skin barrier proteins FLG and TGM and the moisturizing factor HASs has been reported, so we examined the effect of Rb-ME on the expression of these proteins. As a result, we observed that the expression of HAS-2 and -3 was increased in Rb-ME treated cells, and these results suggest that Rb-ME is expected to delay skin aging by improving moisturization.

Other typical symptoms of skin aging include freckles, irregular pigmentation, and age spots. Because this is due to an increase in melanin pigment, the effect of Rb-ME on melanogenesis was evaluated. Rb-ME inhibited melanin production but not secretion. Moreover, Rb-ME reduced the cellular tyrosinase activity involved in melanin production but did not decrease that of purified tyrosinase activity. This implies that tyrosinase is not a direct target of Rb-ME, and further studies have revealed that Rb-ME modulates tyrosinase activity by reducing the expression of tyrosinase, MITF, and TYRP-1 at 200 and 400 *μ*g/ml.

**Figure 6:**
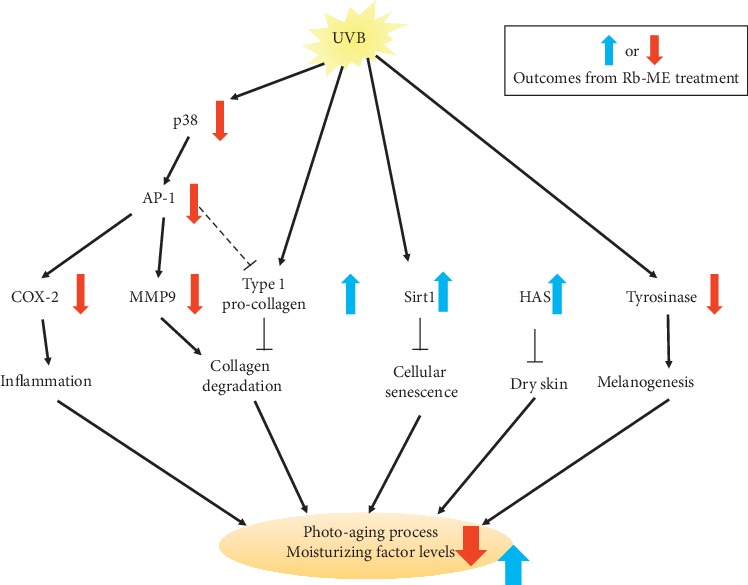
Putative regulatory pathway of Rb-ME mediated antiphotoaging events. UVB-triggered increases in COX-2 and MMP-9 expression, degradation of collagen, and cellular senescence were suppressed by Rb-ME treatment through suppression of the p38/AP-1 pathway. Moisturizing factor expression under normal culture conditions was also elevated by treatment of Rb-ME.

Taken together, these results indicate that Rb-ME has attenuated UVB-induced skin photoaging such as cellular senescence, skin drying, collagen degradation, melanogenesis, and inflammation by regulating p38/AP-1 pathways (Figure 6). Based on these properties, Rb-ME is expected to be used as an active ingredient of cosmetics for antiaging, skin moisturizing, elasticity, and pigmentation alleviation.

## Figures and Tables

**Table 1:** PCR primers used in this study.

Name		Sequence (5′ to 3′)
MMP-9	F	GCCACTTGTCGGCGATAAGG
	R	CACTGTCCACCCCTCAGAGC

Type-1 procollagen	F	CAGGTACCATGACCGAGACG
	R	AGCACCATCATTTCCACGAG

SIRT1	F	CAGTGTCATGGTTCCTTTGC
	R	CACCGAGGAACTACCTGAT

COX-2	F	CAAAAGCTGGGAAGCCTTCT
	R	CCATCCTTCAAAAGGCGCAG

Tyrosinase	F	GTCCACTCACAGGGATAGCAG
	R	AGAGTCTCTGTTATGGCCGA

MITF	F	AACTCATGCGTGAGCAGATG
	R	TACCTGGTGCCTCTGAGCTT

TYRP-1	F	ATGGAACGGGAGGACAAACC
	R	TCCTGACCTGGCCATTGAAC

TYRP-2	F	CAGTTTCCCCGAGTCTGCAT
	R	GTCTAAGGCGCCCAAGAACT

FLG	F	AAGGAACTTCTGGAAAAGGAATTTC
	R	TTGTGGTCTATATCCAAGTGATCCAT

TGM	F	CCCCCGCAATGAGATCTACA
	R	ATCCTCATGGTCCACGTACACA

HAS-1	F	CCACCCAGTACAGCGTCAAC
	R	CATGGTGCTTCTGTCGCTCT

HAS-2	F	TTCTTTATGTGACTCATCTGTCTCACCGG
	R	ATTGTTGGCTACCAGTTTATCCAAACG

HAS-3	F	TATACCGCGCGCTCCAA
	R	GCCACTCCCGGAAGTAAGACT

H.GAPDH	F	GGTCACCAGGGCTGCTTTTA
	R	GATGGCATGGACTGTGGTCA

M.GAPDH	F	ACCACAGTCCATGCCATCAC
	R	CCACCACCCTGTTGCTGTAG

## Data Availability

The data used to support the findings of this study are available from the corresponding author upon request.

## Abbreviations

UV: Ultraviolet
Rb-ME: *Ranunculus bulumei* methanol extract
MAPK: Mitogen-activated protein kinase
ERK: Extracellular response kinase
JNK: c-Jun-N-terminal kinase
HAS: Hyaluronic acid synthase
MMP: Matrix metalloproteinase
MITF: Microphthalmia-associated transcription factor
TYRP: Tyrosinase-related protein
COX-2: Cyclooxygenase-2
AP-1: Activator protein-1
SIRT1: Sirtuin1
RT-PCR: Reverse transcription-polymerase chain reaction
MTT: 3-(4,5-Dimethylthiazol-2-yl)-2,5-diphenyltetrazolium bromide
HPLC: High-performance liquid chromatography
*α*-MSH: *α*-Melanocyte-stimulating hormone
*β*-gal: *β*-Galactosidase
Col1a1: Collagen type I alpha 1
Smad3: Mothers against decapentaplegic homolog 3
PMA: Phorbol-12-myristate-13 acetate
DMEM: Dulbecco's modified eagle's media.
